# Interaction between drinking and dietary inflammatory index affects prostate specific antigen: a cross-sectional study

**DOI:** 10.1186/s12877-023-04151-2

**Published:** 2023-09-05

**Authors:** Xiangtao Weng, Wenyue Tan, Baian Wei, Shijian Yang, Chiming Gu, Shusheng Wang

**Affiliations:** 1 Guangdong Provincial Hospital of Chinese Medicine, Guangzhou, China; 2 The Second Clinical College, Guangzhou University of Chinese Medicine, Guangzhou, China

**Keywords:** Prostate cancer, Dietary inflammatory index, Nutrition, NHANES, Prostate specific antigen

## Abstract

**Background:**

Numerous studies have shown that the dietary inflammatory index (DII) is associated with adverse health effects. However, the relationship between DII and prostate cancer (PCa) remains controversial. Although alcohol is included in DII as a dietary factor, the various adverse health effects of alcohol consumption are not only related to inflammation. On the other hand, it has been a long-standing debate whether alcohol consumption is linked to the risk of PCa. Therefore, to clarify whether drinking affects the relationship between DII and PCa, we evaluated the correlation between DII and prostate-specific antigen (PSA) based on the National Health and Nutrition Examination Survey (NHANES) database.

**Methods:**

We used data from the NHANES spanning from 2005 to 2010 to analyze the relationship between PCa and DII. Out of the 31,034 NHANES participants, we enrolled 4,120 individuals in our study, utilizing dietary intake data from a twenty-four-hour period to determine DII scores. Demographic data, physical and laboratory test results were collected to compare between low PSA and high PSA groups, and to calculate the odds ratio between both groups, we employed a logistic regression analysis.

**Results:**

In this cross-sectional investigation of PCa, drinkers and non-drinkers had different relationships between DII and PSA levels (OR: 1.2, 95% Cl: 1-1.44 vs. OR: 0.98, 95% Cl: 0.9–1.07), and DII and abstaining from alcohol were effective in reducing the incidence of PSA (p-value for significant interaction = 0.037).

**Conclusion:**

The results of our study suggest that drinking may influence the relationship between DII and PSA levels. DII is likely to be a reliable indicator for estimating PSA levels among non-drinkers, who may limit their intake of pro-inflammatory ingredients to lower the incidence and death of PCa.

**Supplementary Information:**

The online version contains supplementary material available at 10.1186/s12877-023-04151-2.

## Introduction

The World Health Organization (WHO) has reported that prostate cancer (PCa) is the second most common cancer among men worldwide [1]. PCa prevalence has escalated into a severe public health problem that leads to growth in related morbidity and mortality, as well as imposing a substantial economic burden [2]. Epidemiological studies have reported significant geographic differences in PCa morbidity and mortality, indicating that lifestyle and dietary factors may play a role in PCa risk [3]. Therefore, identifying the dietary factors associated with PCa is essential in preventing PCa.

It is now recognized that diet plays a significant role in regulating chronic inflammation [4] and that an inflammatory diet is significantly associated with PCa [5].Dietary factors have the potential to influence the microenvironment that regulates PCa cellular proliferation. The Dietary Inflammatory Index (DII) is a novel scoring system that estimates the inflammatory potential of a diet based on quantitative information on dietary intake [6]. To date, DII has been positively associated with cancer incidence and mortality rates globally [7, 8]. On the other hand, Alcohol consumption is associated with various adverse health outcomes and can have a significant impact on health across the lifespan, especially in men [9]. Although alcohol is included in DII as a dietary factor, the various adverse health effects of alcohol consumption are not only related to inflammation. The primary constituent of alcoholic beverages is ethanol, whose metabolism produces acetaldehyde, capable of causing DNA damage, hindering DNA synthesis and repair, and inducing inflammation and oxidative stress, leading to lipid peroxidation [10]. However, Some studies also point out that wine has antioxidant, lipid regulating, and anti-inflammatory effects [11, 12] and alcohol has also been demonstrated to decrease dendritic cell function and increase the inhibitory cytokine IL-10, [13] thus potentially inhibiting the inflammatory pathway.

Currently, the relationship between DII and PCa remains controversial. Several recent studies have pointed to a possible association between DII and PCa risk [14–21]. A recent dose-response meta-analysis suggests that an increased DII is associated with an increased risk of PCa [22]. However, the study by Adriana C. Vidal showed no association between DII and overall or low-grade PCa risk [23]. Similarly, Vázquez-Salas RA did no find a significant association between DII and increased PCa risk [24]. Differences in study results may be attributed to unconsidered potential interactions, such as individual differences, including in prostate-specific antigen (PSA) levels, or other potential factors. PSA is a key biomarker for clinical risk assessment, follow-up, and risk stratification of PCa patients [25, 26]. Therefore, we aim to investigate the association between DII and PSA levels in individuals using the data from the National Health and Nutrition Examination Survey (NHANES) from 2005 to 2010.

## Materials and methods

### Study population and design

**Fig. 1:**
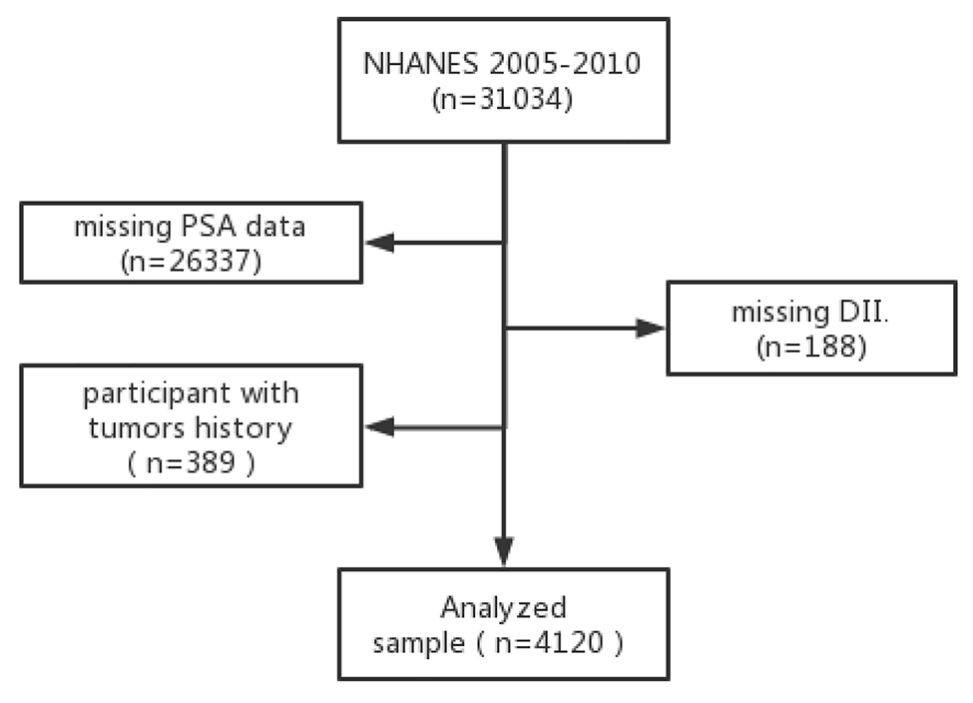
Flowchart of procedures from identification to the inclusion of eligible patients

The National Health and Nutrition Examination Survey (NHANES) is a cross-sectional survey of the American population that collects information on demographics, socioeconomics, food, and health via interviews. NHANES data from 2005–2006, 2007–2008, and 2009–2010 were examined. All participants provided informed consent, and the NHANES technique has been authorized by the US National Center for Health Statistics Research Ethics Review Board. The independent variable of interest was the DII, which was calculated by adding the scores from each component of the food taken on the first day. Covariates included population and physical examination variables, while the dependent variable was the PSA level. Logistic regression models were used to examine differential distributions of the covariates. Our study included males over 40 years old and excluded participants who had incomplete PSA data (n = 26,337), lacked DII data (n = 188), or had a history of tumors (n = 4,726). Overall, the study included 4,120 participants. See Fig. 1 for a detailed flowchart. More information on NHANES can be found at https://www.cdc.gov/nchs/nhanes/default.aspx.

### Data availability

Since 1960, the National Center for Health Statistics (NCHS) has been conducting the NHANES survey every two years to assess the health and nutritional status of the noninstitutional population in the United States (CDC, 2021). The NHANES survey is authorized by the NCHS study ethical review board, and written informed consent is obtained from all participants. Additional details about NHANES can be found on the official NHANES website at https://www.cdc.gov/nchs/nhanes/.

### Variables

#### Calculation of the DII

DII is a grading system developed by Shivappa to evaluate the potential inflammatory effects of various dietary components through a comprehensive literature analysis. The DII consists of 45 ingredients that are used to calculate the impact of dietary consumption on inflammation. To calculate the DII, the scores for each dietary component consumed over 24 h, including both pro-inflammatory and anti-inflammatory diet scores, are added together. Briefly, the Z score is obtained by subtracting the Global Daily Mean Intake, dividing by the Standard Deviation and then converting the result to a percentile score. Each percentile score is subsequently doubled and one is subtracted to create a symmetrical distribution. By adding up each DII score and obtaining the percentile value, an individual’s overall DII score can be determined, which is then multiplied by the matching overall inflammatory impact score [6].

The DII is a grading system Shivappa created after doing a literature analysis to assess the potential inflammatory levels of dietary components. The DII uses 45 ingredients to calculate the impact of dietary consumption on inflammation. The DII is calculated by adding the scores from each dietary component that was consumed over the course of 24 h, including the pro-inflammatory and anti-inflammatory diet scores. Briefly stated, the Z score is a value created by subtracting the Global Daily Mean Intake, dividing by the Standard Deviation, and finally converting the result to a percentile score. Finally, each percentile score is doubled and a “1” is subtracted to create a symmetrical distribution. We may determine an individual “overall DII score” by adding up each DII score to get the percentile value, which is then multiplied by the matching “overall inflammatory impact score.“Briefly stated, the Z score is a value created by subtracting the Global Daily Mean Intake, dividing by the Standard Deviation, and finally converting the result to a percentile score. Finally, each percentile score is doubled and a “1” is subtracted to create a symmetrical distribution. We may determine an individual “overall DII score” by adding up each DII score to get the percentile value, which is then multiplied by the matching “overall inflammatory impact score” [6].

The NHANES 2005–2010 dataset comprised 28 food parameters that were utilized to calculate DII. These food parameters involved alcohol, cholesterol, carbohydrates, energy, fiber, iron, magnesium, zinc, selenium, carotene, vitamins A, B6, B12, C, D, and E, monounsaturated fatty acids, protein, niacin, riboflavin, fats, folic acid, and omega-6 and omega-3 fatty acids.

#### PSA

For the current study, serum PSA concentration, measured in ng/mL, was determined using the Hybritech Total PSA Assay in the Beckman Access Immunoassay System (Beckman Coulter, Fullerton, CA) [27]. All male participants above 40 years were eligible to have their PSA levels assessed, except those with recent rectal examination within the preceding week, cystoscopy or prostate biopsy within the past month, ongoing prostate gland inflammation or infection, and any history of PCa [28]. Dichotomous ( < = 4 ng/mL or > 4 ng/mL) PSA data were used as outcome variables in our analyses. where 4 ng/mL is the current clinical cut-point for a positive screen [29–31].

#### Covariates

The selection of covariates was determined based on a combination of previously published studies, clinical experience, pathophysiological analysis, and pragmatic considerations for future clinical practice. Various confounding factors, including demographic and survey-related factors, were readily apparent in the database. These confounders included continuous variables such as age in years and Body Mass Index (BMI) in kg/m2. Categorical factors, on the other hand, included race, education level, marital status, history of high blood pressure, diabetes, heart attack and stroke, Metabolic Syndrome (MetS), smoking, and physical activity.

The diagnosis of the Metabolic Syndrome (Mets) was based on Adult Treatment Panel III criteria, [32] while the diagnosis of diabetes was determined by taking into account six factors: (1) self-reported diagnosis by a doctor of diabetes (DIQ010); (2) glycohemoglobin HbA1c (percent) greater than 6.5 (LBXGH); (3) fasting glucose levels (mmol/L) greater than or equal to 7.0 (LBDGLUSI); (4) random blood glucose levels (mmol/L) greater than or equal to 11.1 (LBDSGLSI); (5) two-hour OGTT blood glucose levels (mmol/L) greater than or equal to 11.1 (LBXGLT); and (6) self-reported use of diabetes medication (RXQ_DRUG). Participants with diabetes were categorized into one of three groups: those with Diabetes Mellitus (DM), those with Impaired Fasting Glucose (IFG), those with Impaired Glucose Tolerance (IGT), and those without diabetes. Smokers were categorized into one of the following three groups: never smoked more than 100 cigarettes in their lifetime (SMQ020), former smokers who had smoked more than 100 cigarettes in their lifetime but were currently not smoking (SMQ020), and current smokers who had smoked more than 100 cigarettes in their lifetime and were currently smoking (SMQ040). Alcohol consumption was evaluated using the MCQ questionnaire, which asked participants if they had consumed at least 12 alcoholic beverages in the past year (ALQ101). Physical activity levels were quantified as metabolic equivalents (MET) and were classified as inactivity, low activity, and high activity for those with 0, 0.1–7.5, and > = 7.5 MET-h/week, respectively. Participants who reported ever being told by a healthcare professional that they had experienced a stroke were grouped as having had a stroke (MCQ160f). Hypertension was classified as present or absent, based on the following conditions: (1) ever being diagnosed with high blood pressure (BPQ020) or being told they had it twice or more (BPQ030), (2) self-reported use of hypertension medication (BPQ040a, RXQ_DRUG), and (3) systolic blood pressure measurements > = 140 mmHg or diastolic blood pressure measurements > = 90 mmHg recorded three times (BPXSY, BPXDI). Further details on these factors can be obtained from the NHANES website.

### Statistical analysis

The recommended statistical analysis procedures of the Centers for Disease Control and Prevention (CDC) were adhered to. The source of these procedures is https://wwwn.cdc.gov/nchs/nhanes/tutorials/default.aspx.

for increased statistical power. The PSA data were split into two groups based on a threshold of 4ng/mL to enhance statistical power. Multivariable logistic regression analysis was utilized to investigate PSA levels. Extended logistic models were employed to control for several variables. Participants underwent a descriptive analysis. We presented the categorical variables as proportions (in percent) and computed the means and standard deviations (SD) or medians and interquartile ranges (IQR) for continuous data. We performed one-way ANOVA, Kruskal-Wallis, and chi-square tests (for categorical variables) to compare the variables. We employed logistic regression models, which included the DII and other relevant factors, for subgroup analysis. Drinking was the stratification factor (i.e., drinkers and non-drinkers). We also explored how DII interacted by including an interaction term in the model that multiplied the two predictor variables. Furthermore, we divided DII into three groups based on triquantiles to test for linear trends. Subsequently, the median value of each DII category was included as a continuous variable in the models.

We carried out the statistical analysis using R version 4.2.1 (http://www.R-project.org, The R Foundation) and Free Statistics software version 1.7. We conducted a two-tailed test, and a p-value less than 0.05 was deemed statistically significant.

## Results

### Baseline characteristics of the study population

Table 1 presents the general characteristics of the study population based on the PSA level. Individuals with high PSA (> 4 ng/mL) were more likely to be older, non-drinkers, have a higher DII, higher energy intake and a history of heart attacks, strokes, and hypertension, relative to those with low PSA ( < = 4 ng/mL). People with high PSA levels had poor BMI values and were more likely to have a sedentary lifestyle or low activity. PSA level did not differ based on PIR, education, smoking habits, diabetes, or Metabolic Syndrome (MetS) history.

**Table 1:** Baseline characteristics of selected participants

Variables	Total (n = 4120)	PSA < = 4 (n = 3832)	PSA > 4 (n = 288)	p
**Age, Mean ± SD**	58.4 ± 12.2	57.6 ± 12.0	69.3 ± 9.4	< 0.001
**Marry, n (%)**				< 0.001
Divorced	487 (11.8)	458 (12)	29 (10.1)	
Living with partner	224 ( 5.4)	211 (5.5)	13 (4.5)	
Married	2740 (66.6)	2559 (66.8)	181 (62.8)	
Never married	303 ( 7.4)	284 (7.4)	19 (6.6)	
Separated	125 ( 3.0)	118 (3.1)	7 (2.4)	
Widowed	238 ( 5.8)	199 (5.2)	39 (13.5)	
**PIR, n (%)**				0.663
<1	632 (16.6)	585 (16.5)	47 (17.5)	
>=1	3179 (83.4)	2958 (83.5)	221 (82.5)	
**Education, n (%)**				0.827
<High school	667 (16.2)	617 (16.1)	50 (17.4)	
High school	1596 (38.7)	1484 (38.7)	112 (38.9)	
>High school	1856 (45.1)	1730 (45.2)	126 (43.8)	
**BMI, Mean ± SD**	29.0 ± 5.8	29.1 ± 5.9	28.0 ± 5.2	0.002
**Race, n (%)**				0.008
White	2072 (50.3)	1916 (50)	156 (54.2)	
Black	822 (20.0)	760 (19.8)	62 (21.5)	
Mexican	735 (17.8)	705 (18.4)	30 (10.4)	
other	491 (11.9)	451 (11.8)	40 (13.9)	
**Smoke, n (%)**				0.059
never	1626 (39.5)	1506 (39.3)	120 (41.7)	
former	1545 (37.5)	1427 (37.2)	118 (41)	
now	948 (23.0)	898 (23.4)	50 (17.4)	
**Drinker, n (%)**				0.006
No	669 (17.1)	606 (16.6)	63 (23.1)	
Yes	3249 (82.9)	3039 (83.4)	210 (76.9)	
**Stroke, n (%)**				0.022
No	3897 (94.9)	3633 (95.1)	264 (92)	
Yes	211 ( 5.1)	188 (4.9)	23 (8)	
**MetS, n (%)**				0.211
No	2591 (62.9)	2400 (62.6)	191 (66.3)	
Yes	1529 (37.1)	1432 (37.4)	97 (33.7)	
**Diabetes, n (%)**				0.628
No	2656 (64.5)	2480 (64.7)	176 (61.1)	
IFG	275 ( 6.7)	255 (6.7)	20 (6.9)	
IGT	191 ( 4.6)	175 (4.6)	16 (5.6)	
DM	998 (24.2)	922 (24.1)	76 (26.4)	
**Hypertension, n (%)**				< 0.001
No	1971 (47.8)	1868 (48.7)	103 (35.8)	
Yes	2149 (52.2)	1964 (51.3)	185 (64.2)	
**Heart attack, n (%)**				0.009
No	3776 (91.8)	3525 (92.1)	251 (87.8)	
Yes	336 ( 8.2)	301 (7.9)	35 (12.2)	
**Activity, n (%)**				< 0.001
Inactive	1098 (26.7)	1002 (26.1)	96 (33.3)	
Low-active	693 (16.8)	632 (16.5)	61 (21.2)	
Highly active	2329 (56.5)	2198 (57.4)	131 (45.5)	
**DII, Mean ± SD**	1.3 ± 1.9	1.2 ± 1.9	1.5 ± 1.9	0.026
**Energy(cal), Mean ± SD**	2323.2 ± 1044.6	2348.5 ± 1053.2	1987.5 ± 856.0	< 0.001

PSA:prostate specific antigen; DII, Dietary Inflammatory Index; PIR,Poverty Impact Ratio; MetS, Metabolic Syndrome;IFG, Impaired fasting glucose; IGT, Impaired glucose tolerance; DM, Diabetes mellitus.

### Distribution of DII in the PSA group by drinker

**Fig. 2:**
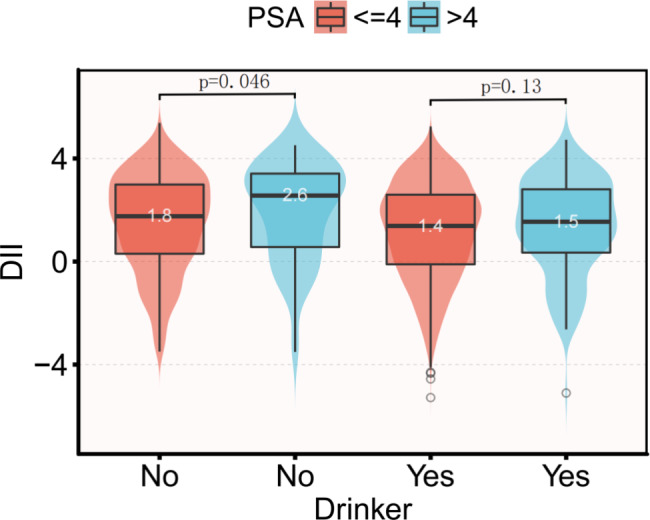
Distribution of DII in patients with higher PSA grouped by drinker

Figure 2 illustrates the comparison of DII levels between participants with high and low PSA levels. However, in the group of drinkers, the difference was not statistically significant (1.4 vs. 1.5, p = 0.13). In contrast, among the non-drinker group, participants with high PSA had significantly higher DII values compared to those with low PSA (1.8 vs. 2.6, p = 0.046).

### Drink habit affects the association between DII and PSA Level

The univariate analysis showed that age, BMI, marital status, race, exercise intensity, energy, and history of stroke, hypertension, and heart attack were significantly associated with PSA level (Table 2). Upon adjusting for age, marital status, education, PIR, BMI, race, smoking habit, and history of stroke, MetS, diabetes, hypertension, heart attack, physical activity, and drinking status, a significant interaction effect was found between DII and PSA level (Table 3). This interaction has also been detected in the other three distinct adjustment models (Supplementary Tables S1–S3).

The analysis showed that drinking had a significant impact on the association between PSA and DII (p-value for the interaction likelihood ratio test was 0.037), where a significant increase in PSA was observed as DII increased in the non-drinker group (p = 0.049). However, no significant difference was observed in the group of drinkers (p = 0.676). After triquantile transformation, a significant interaction between DII and PSA level was observed for both drinkers and non-drinkers (the interaction likelihood ratio test’s p-value was 0.023).

**Table 2:** Association of covariates and PSA level-up risk

Variable	OR_95CI	P_value
**Age**	1.09 (1.07 ~ 1.1)	< 0.001
**BMI**	0.96 (0.94 ~ 0.98)	< 0.001
**PIR, n (%)**		
1<	1(reference)	
>=1	0.85 (0.62 ~ 1.15)	0.293
**Marry, n (%)**		
Divorced	1(reference)	
Living with partner	0.89 (0.46 ~ 1.73)	0.735
Married	1.05 (0.71 ~ 1.54)	0.812
Never married	1.05 (0.59 ~ 1.86)	0.864
Separated	0.85 (0.37 ~ 1.97)	0.705
Widowed	2.95 (1.82 ~ 4.79)	< 0.001
**Education, n (%)**		
<High School	1(reference)	
High school	0.93 (0.66 ~ 1.32)	0.687
>High school	0.9 (0.64 ~ 1.26)	0.539
**Race, n (%)**		
Mexican	1(reference)	
White	1.72 (1.18 ~ 2.52)	0.005
Black	1.76 (1.15 ~ 2.7)	0.009
Other	1.93 (1.22 ~ 3.07)	0.005
**Smoke, n (%)**		
former	1(reference)	
never	0.97 (0.75 ~ 1.25)	0.803
now	0.73 (0.52 ~ 1.01)	0.054
**Stroke, n (%)**		
no	1(reference)	
yes	1.68 (1.08 ~ 2.62)	0.021
**MetS, n (%)**		
no	1(reference)	
yes	0.8 (0.61 ~ 1.05)	0.102
**Diabetes, n (%)**		
no	1(reference)	
DM	1.18 (0.9 ~ 1.54)	0.24
IFG	1.12 (0.7 ~ 1.78)	0.648
IGT	1.26 (0.74 ~ 2.14)	0.399
**Hypertension, n (%)**		
no	1(reference)	
yes	1.74 (1.36 ~ 2.21)	< 0.001
**Heart attack, n (%)**		
no	1(reference)	
yes	1.59 (1.1 ~ 2.29)	0.014
**Activity, n (%)**		
inactive	1(reference)	
low-active	1.05 (0.75 ~ 1.47)	0.768
highly active	0.65 (0.5 ~ 0.84)	0.001
**DII**	1.08 (1.01 ~ 1.15)	0.026
**Energy**	1 (1 ~ 1)	< 0.001
**Drinker, n (%)**		
No	1(reference)	
Yes	0.63 (0.47 ~ 0.83)	0.001

DII, Dietary Inflammatory Index; PIR,Poverty Impact Ratio; MetS, Metabolic Syndrome;IFG, Impaired fasting glucose; IGT, Impaired glucose tolerance; DM, Diabetes mellitus.

**Table 3:** Interaction of drinking and DII on PSA level up

variable	Non-drinker (n = 669)			Drinker (n = 3249)		P for interaction
	OR(95% CI)	P-value		OR(95% CI)	P-value	
DII	1.2 (1 ~ 1.44)	0.049		0.98 (0.9 ~ 1.07)	0.676	0.037
Subgroups						0.023
Higher(2.27 ~ 5.38)	1(Ref)			1(Ref)		
Middle(0.51 ~ 2.27)	0.47 (0.22 ~ 1.04)	0.061		1.1 (0.76 ~ 1.61)	0.605	
Low(-5.28 ~ 0.51)	0.57 (0.26 ~ 1.25)	0.16		0.94 (0.63 ~ 1.41)	0.766	
Trend test	1.38 (0.93 ~ 2.06)	0.108		1.03 (0.84 ~ 1.25)	0.786	

Adjusted for age, race/ethnicity, marital status, PIR, education, BMI, activity, and the history of stroke, MetS, diabetes, hypertension, and heart attack.

## Discussion

We made use of the NHANES database, which contains data on the dietary habits of Americans. Our study shows that the PSA in the non-drinker group increased as the DII increased, indicating that the DII is probably a helpful index for PCa patients to use to direct their diet. Although there is no difference in significance between subgroups, the drink group’s p-value is higher and its OR is lower than that of the non-drinkers, indicating that non-drinkers may benefit from an anti-inflammatory diet in preventing PCa.

An elevated risk of chronic diseases like cancer has been related to persistent low-grade systemic inflammation [33]. There is evidence linking systemic and prostatic inflammation to prostate tumorigenesis [34–37]. Particularly, in both benign and malignant prostatic diseases, an inflammatory milieu may stimulate cellular proliferation [38]. Besides, the majority of pro- and anti-inflammatory chemicals come from food [39]. To determine its general propensity for inflammation, DII was developed. It is based on a thorough review of the literature that includes studies on how nutrition affects inflammation in cell culture, animals, and epidemiology [6]. To test the hypothesis that dietary inflammation affects the risk and mortality of non-communicable diseases, the DII has undergone considerable research in a number of disease situations since its inception [40]. Recently, a meta-analysis revealed that across cancer types, research populations, and study design, there were consistent and substantial positive correlations between increased DII with cancer incidence and mortality [7]. Additionally, a different meta-aggregate utilizing credibility evaluation revealed a strong correlation between DII and the risk of dying from any cause, the risk of dying from cancer overall, and site-specific malignancies [8].

However, the current study finds that the association between DII and PCa is still controversial. Among the studies that previously assessed the association between DII and PCa risk, ten were case-control studies [14–20, 23, 24, 41] and two were prospective studies [21, 42]. Higher levels of inflammatory biomarkers are positively correlated with an increased risk of PCa, according to several research [14–21]. A relationship between higher DII and an increased risk of PCa was also found in a dose-based meta-analysis study conducted in 2020 by Zhu Y et al. [22] This is reasonably close to what we found. However, other experts also point out that there is no correlation between increased DII and total PCa risk [23, 24, 41, 42]. Interestingly, the California Men’s Health Study (CMHS) prospective cohort of 40,161 men discovered a relationship between a higher DII (3rd Q) and a higher risk of high-grade PCa, but this association vanished in the 4th Q, p-trend = 0.74, indicating a non-linear dose-response relationship. Our results also revealed this non-linear relationship in the subgroup analysis (p for trend > 0.05) [42].

The slight disparity between the several research could be explained by a number of factors. The different studies employed different meal factors to determine DII ratings. Additionally, different foods and beverages are included in the FFQ depending on the location. Furthermore, various study populations also have distinct effects [23].Despite the fact that dietary patterns vary greatly around the world, the data that are now accessible are primarily from cultures with pro-inflammatory eating habits. But a case-control study in the Vietnamese community similarly supported the idea that elevated levels of inflammatory biomarkers are associated with a higher risk of PCa [18]. Overall, higher DII scores indicate a more pro-inflammatory diet is linked to higher incidence risks for PCa. Additionally, a prospective research of French people in their middle age discovered that DII was linked to a higher risk of developing PCa and that the combination of DII and alcohol consumption (g/d) was related with a lower overall risk of developing cancer (P-interaction = 0.02) [21]. This further confirms our findings that the non-drink group’s interaction with DII had an impact on PSA. Additionally, the interaction may be more effective in directing the application of DII to non-drinking populations in order to delay the onset of PCa.

Our research has several limitations. The correlations found in cross-sectional observation study may not indicate direct causality, to begin with, and they may be muddled by other unmeasured factors. However, a number of potential confounders, such as age and race, were adjusted in the logistic regression model. Second, even with such a sizable sample, the research population was limited to US nationals and the calculation of the DII is obtained using the data of dietary intakes during a twenty-four-hour period, which should be considered when extrapolating to other populations. Additionally, even though the interaction test is significant, neither the drinker nor non-drinker groups in the subgroup analysis demonstrate a significant relationship between the DII groups and PSA level. Furthermore, remembrance bias and self-reporting bias may cause the results from self-reported 24-hour dietary recalls to be biased. An inaccurate result may be obtained if the same person were resampled in a different district. As a result, each year the NHANES examined over 5,000 people in 15 distinct counties across the nation. Participants were chosen using a multistage, stratified probability approach. Finally, PSA may be controversial with this test due to the chance of false positive results. The PSA testing guidelines are regularly updated to include subgroups of men who might benefit from the testing the most while experiencing the fewest negative effects [43]. As part of this revision to the recommendations, the previously recommended cutoff threshold of 4 ng/ml might be altered [44]. We continue to use the 4 ng/ml cutoff number in this inquiry based on previous recommendations. If the indicated PSA cutoff level is changed, the therapeutic significance of these data might be limited. It should be important to corroborate our findings with additional carefully planned multi-center controlled studies in light of these limitations.

## Conclusion

The results of our study indicated that drinking might affect the association of DII with the PSA level. DII is likely to be a good index for non-drinkers to predict the PSA level. Although, DII data were obtained from 24-hour dietary recall interview-derived or food record data, but it can be used with dietary data from any source and be used for guiding non-drinkers in setting dietary goals to reduce their intake of pro-inflammatory, which possibly reduce PCa incidence and mortality. Besides, dietary intervention might be a promising method in the therapy of prostate for non-drinkers. Although we had offered some clinical hints, further randomized controlled research is required in the future to offer more proof.

### Electronic supplementary material

Below is the link to the electronic supplementary material.

Supplementary Material 1

## Data Availability

The datasets generated and analyzed during the current study are available in the NHANES website, available at https://www.cdc.gov/nchs/nhanes/index.htm.
