# Supplementary material for: Interaction between drinking and dietary inflammatory index affects prostate specific antigen: a cross-sectional study
Source: BMC Geriatr. 2023 Sep 5;23:537. doi: 10.1186/s12877-023-04151-2 (PMC10478225; doi:10.1186/s12877-023-04151-2)
Supplement: Supplementary file 1 — Supplementary Material 1 [file 12877_2023_4151_MOESM1_ESM.docx]

| TABLE S1 Interactive effect of DII and drinking on PSA level (Model 1) | | | | | | |
| --- | --- | --- | --- | --- | --- | --- |
| variable | Non-drinker  (n=669) | |  | Drinker  (n=3249) | | P for interaction |
|  |  |  |  |  |  |  |
|  | OR(95% CI) | P-value |  | OR(95% CI) | P-value |  |
| DII |  |  |  |  |  | 0.045 |
| higher | 1(Ref) |  |  | 1(Ref) |  |  |
| middle | 0.46 (0.24~0.9) | 0.023 |  | 0.97 (0.7~1.35) | 0.853 |  |
| low | 0.63 (0.34~1.19) | 0.154 |  | 0.71 (0.5~1.02) | 0.062 |  |
| Trend.test | 1.33 (0.96~1.84) | 0.091 |  | 1.18 (0.99~1.4) | 0.064 |  |
| No covariate was adjusted. | | | | | | |

| TABLE S2 Interactive effect of DII and drinking on PSA level (Model 2) | | | | | | |
| --- | --- | --- | --- | --- | --- | --- |
| variable | Non-drinker  (n=669) | |  | Drinker  (n=3249) | | P for interaction |
|  |  |  |  |  |  |  |
|  | OR(95% CI) | P-value |  | OR(95% CI) | P-value |  |
| DII |  |  |  |  |  | 0.032 |
| higher | 1(Ref) |  |  | 1(Ref) |  |  |
| middle | 0.49 (0.25~0.99) | 0.048 |  | 1.16 (0.82~1.64) | 0.406 |  |
| low | 0.63 (0.33~1.22) | 0.173 |  | 0.95 (0.66~1.38) | 0.8 |  |
| Trend.test | 1.32 (0.94~1.85) | 0.115 |  | 1.02 (0.85~1.22) | 0.845 |  |
| Adjusted for age, race/ethnicity. | | | | | | |

| TABLE S3 Interactive effect of DII and drinking on PSA level (Model 3) | | | | | | |
| --- | --- | --- | --- | --- | --- | --- |
| variable | Non-drinker  (n=669) | |  | Drinker  (n=3249) | | P for interaction |
|  |  |  |  |  |  |  |
|  | OR(95% CI) | P-value |  | OR(95% CI) | P-value |  |
| DII |  |  |  |  |  | 0.028 |
| Higher(2.27~5.38) | 1(Ref) |  |  | 1(Ref) |  |  |
| Middle(0.51~2.27) | 0.46 (0.22~0.98) | 0.043 |  | 1.1 (0.76~1.59) | 0.631 |  |
| Low(-5.28~0.51) | 0.55 (0.26~1.15) | 0.112 |  | 0.93 (0.62~1.38) | 0.707 |  |
| Trend.test | 1.42 (0.97~2.08) | 0.073 |  | 1.04 (0.85~1.26) | 0.728 |  |
| Adjusted for age, race/ethnicity, marital status, PIR, education, BMI and activity. | | | | | | |
